# Supplementary material for: Flash healing of laser-induced graphene
Source: Nat Commun. 2024 Apr 4;15:2925. doi: 10.1038/s41467-024-47341-1 (PMC10995154; doi:10.1038/s41467-024-47341-1)
Supplement: Supplementary file 1 — Supplementary Information [file 41467_2024_47341_MOESM1_ESM.pdf]

Supplementary Information for

Flash Healing of Laser-induced Graphene

Le Cheng,[1,2] *Chi Shun Yeung,*[1,2] *Libei Huang,*[1,3] *Ge Ye,*[1] *Jie Yan,*[4] *Wanpeng Li,*[4] *Chunki Yiu,*[5] *Fu-Rong Chen,*[4] *Hanchen Shen,*[6] *Ben Zhong Tang,*[6] *Yang Ren,*[7,8] *Xinge Yu,*[5*] *Ruquan Ye*[1,2*]

[1]Department of Chemistry, State Key Laboratory of Marine Pollution, City University of Hong Kong, Hong Kong 999077, P. R. China

[2]City University of Hong Kong Shenzhen Research Institute, Shenzhen, Guangdong 518057, P. R. China

[3]Division of Science, Engineering and Health Study, School of Professional Education and Executive Development (PolyU SPEED), The Hong Kong Polytechnic University, Hong Kong 999077, P. R. China

[4]Department of Materials Science and Engineering, Time-resolved Aberration Corrected Environmental Electron Microscope Unit, City University of Hong Kong, Hong Kong 999077, P. R. China

[5]Department of Biomedical Engineering, City University of Hong Kong, Hong Kong 999077, P. R. China

[6]School of Science and Engineering, Shenzhen Institute of Aggregate Science and Technology, The Chinese University of Hong Kong, Shenzhen (CUHK-Shenzhen), Guangdong 518172, P. R. China.

[7]Department of Physics, City University of Hong Kong, Hong Kong 999077, P. R. China

[8]Centre for Neutron Scattering, City University of Hong Kong, Kowloon, Hong Kong 999077, P. R. China

*Corresponding authors.
E-mail: ruquanye@cityu.edu.hk; xingeyu@cityu.edu.hk.

Supplementary Figures

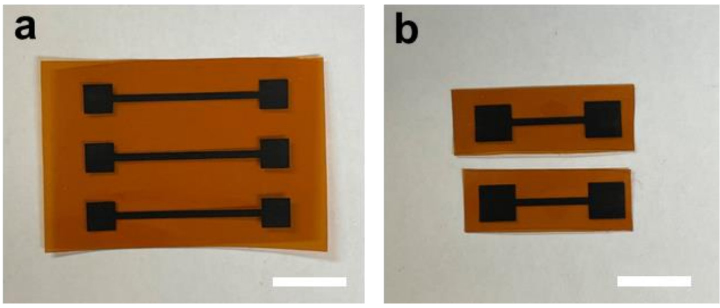

Supplementary Figure 1. Digital photographs of dumbbell shaped LIG patterns. LIG patterns with effective region measuring (a) 1 mm × 20 mm and (b) 1 mm × 10 mm. Scale bars equal 1 cm.

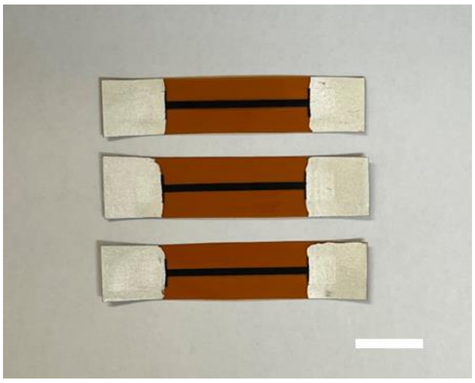

Supplementary Figure 2. Digital photographs of dumbbell shaped LIG patterns with silver paste applied to the ends. Scale bar equals 1 cm.

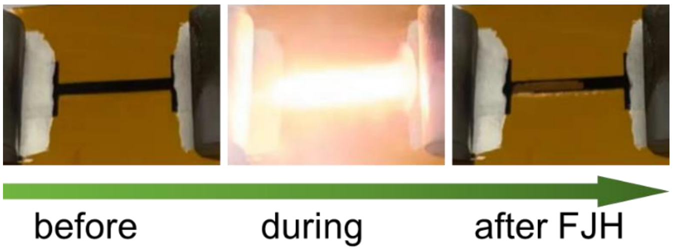

Supplementary Figure 3. Digital photographs of the LIG pattern before, during and after the FJH treatment under 200 V 20 ms.

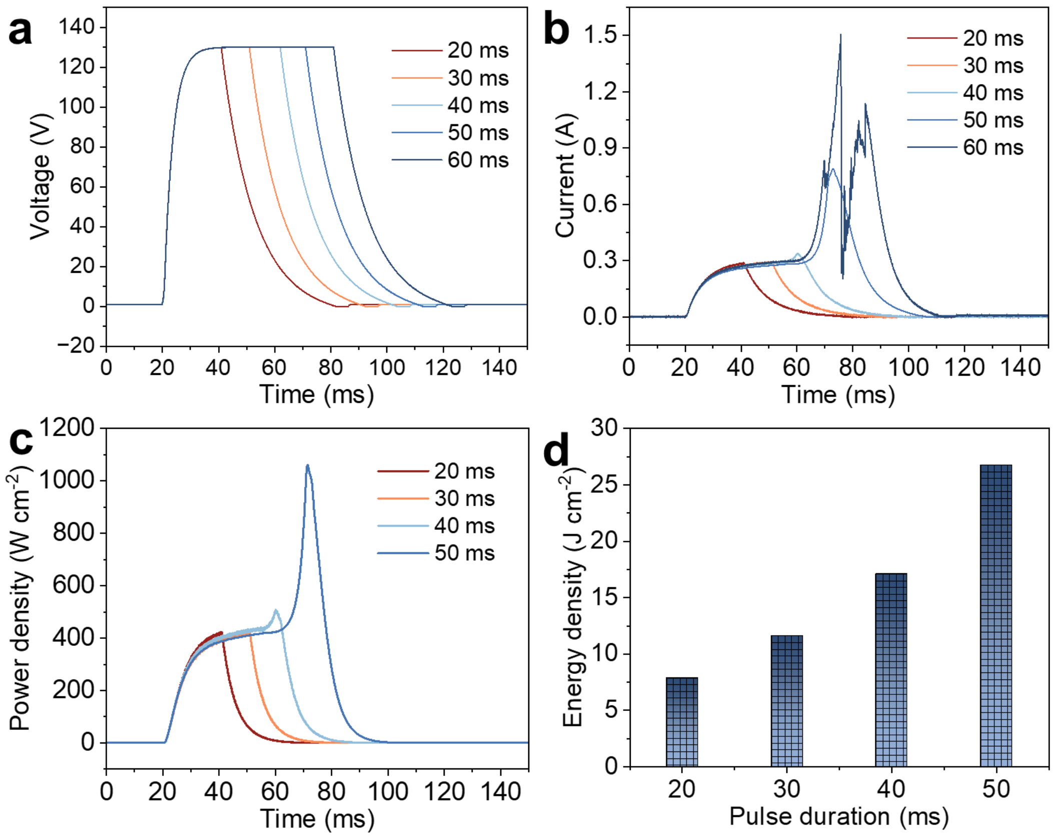

Supplementary Figure 4. Investigation of the FJH process with different pulse durations. (a) Voltage, (b) current, and (c) areal power density profiles, as well as (d) areal energy density during the FJH process of 130 V with various pulse durations.

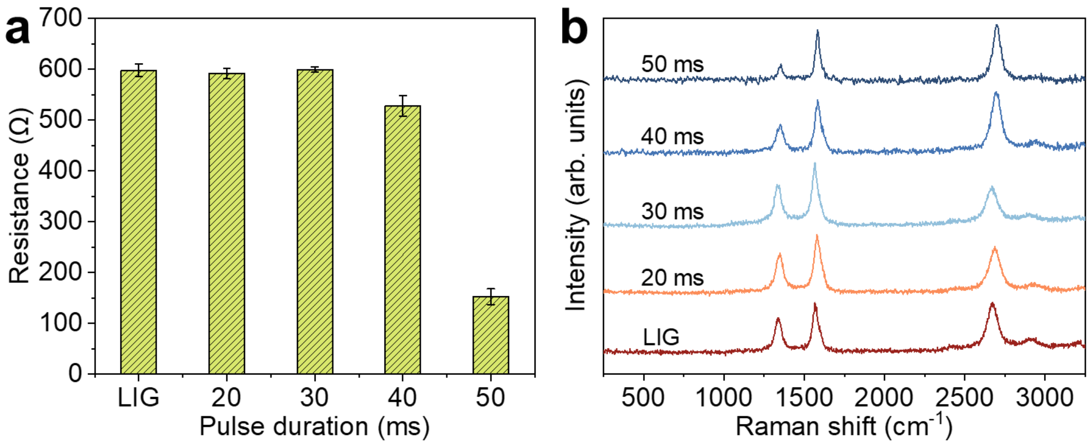

Supplementary Figure 5. Effect of the FJH treatment with different pulse durations. (a) Resistance and (b) Raman spectra of LIG patterns (1 mm × 10 mm) treated under 130 V with different pulse durations. Error bars represent the standard deviation of three independent measurements.

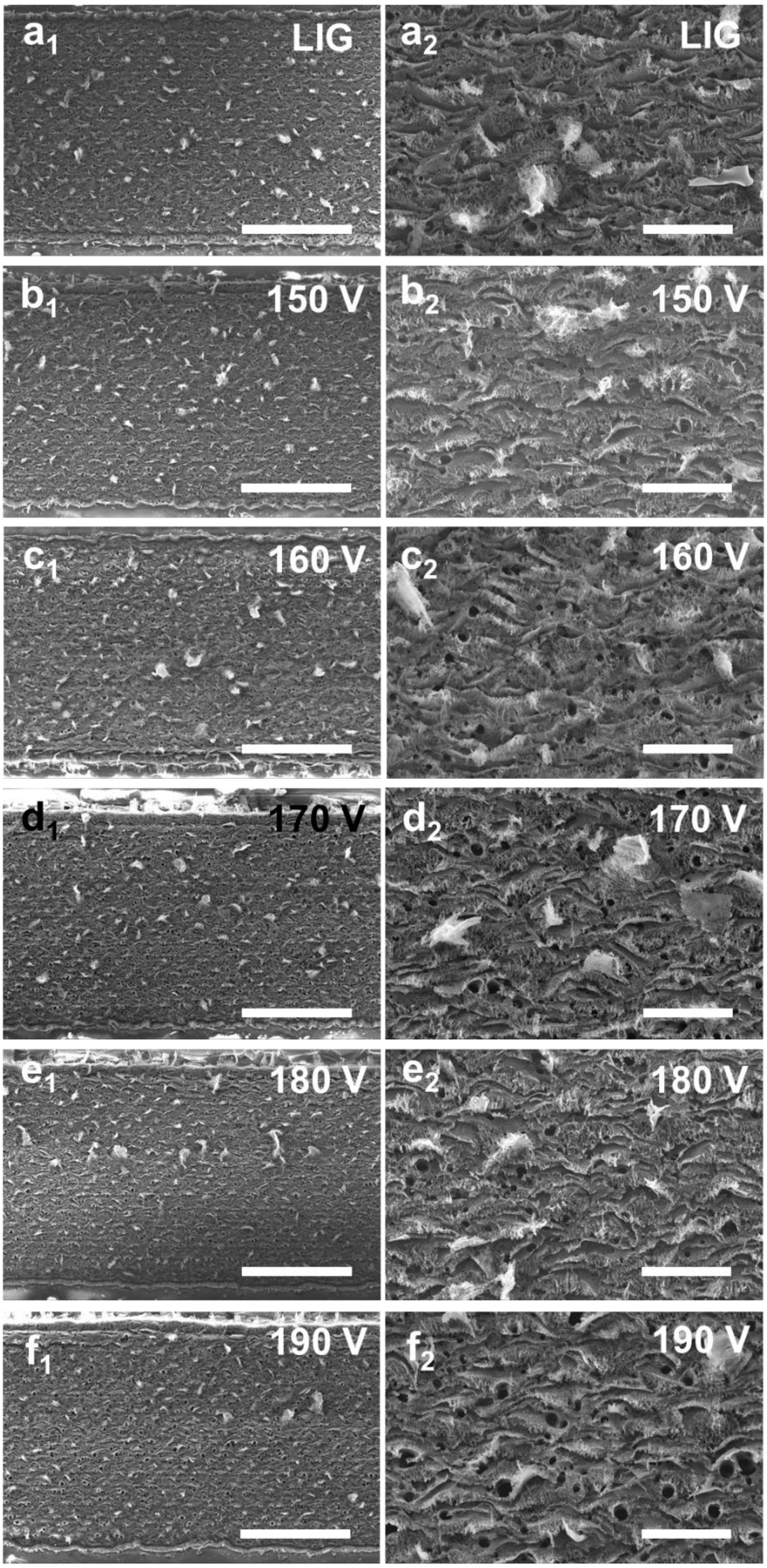

Supplementary Figure 6. Microstructure characterization. SEM images of (a) LIG and (b-f) F-LIG patterns treated under different voltages. Scale bars equal to 500 μm and 100 μm in the normal and enlarged areas, respectively.

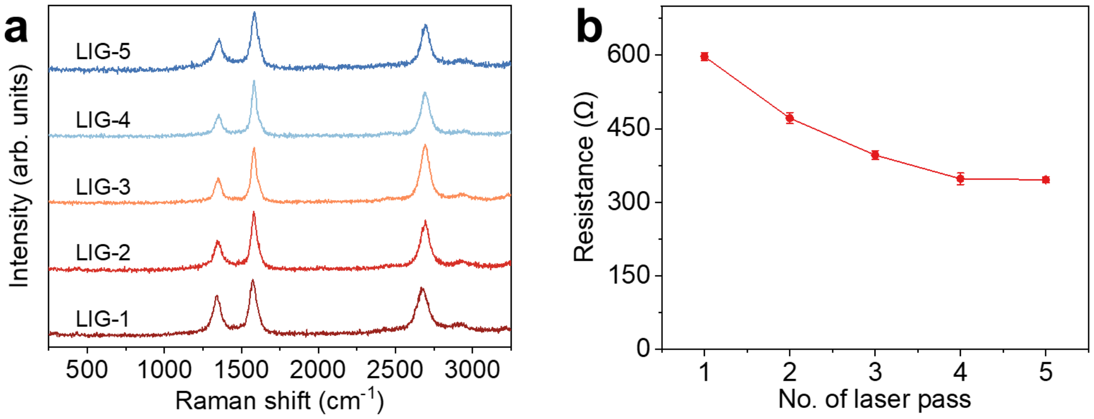

Supplementary Figure 7. Effect of multiple laser passes. (a) Raman spectra and (b) resistance of LIG with different lasing passes. Error bars represent the standard deviation of three independent measurements.

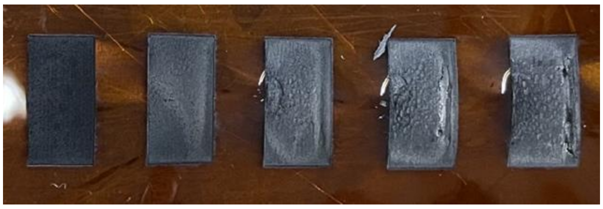

Supplementary Figure 8. Digital photographs of LIG with different laser passes of 1-5 (from left to right).

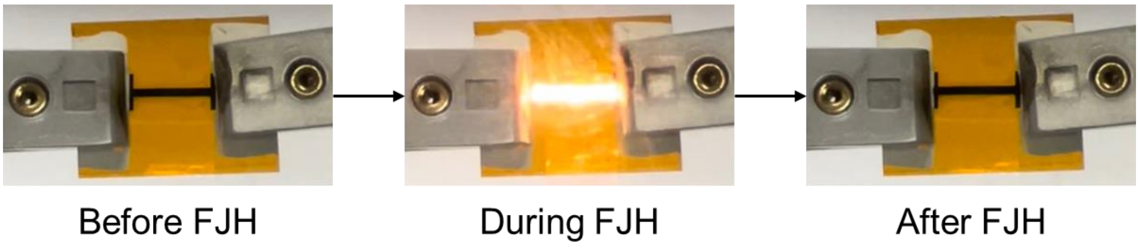

Supplementary Figure 9. Digital photographs of the LIG pattern before, during and after the FJH treatment under 180 V 20 ms.

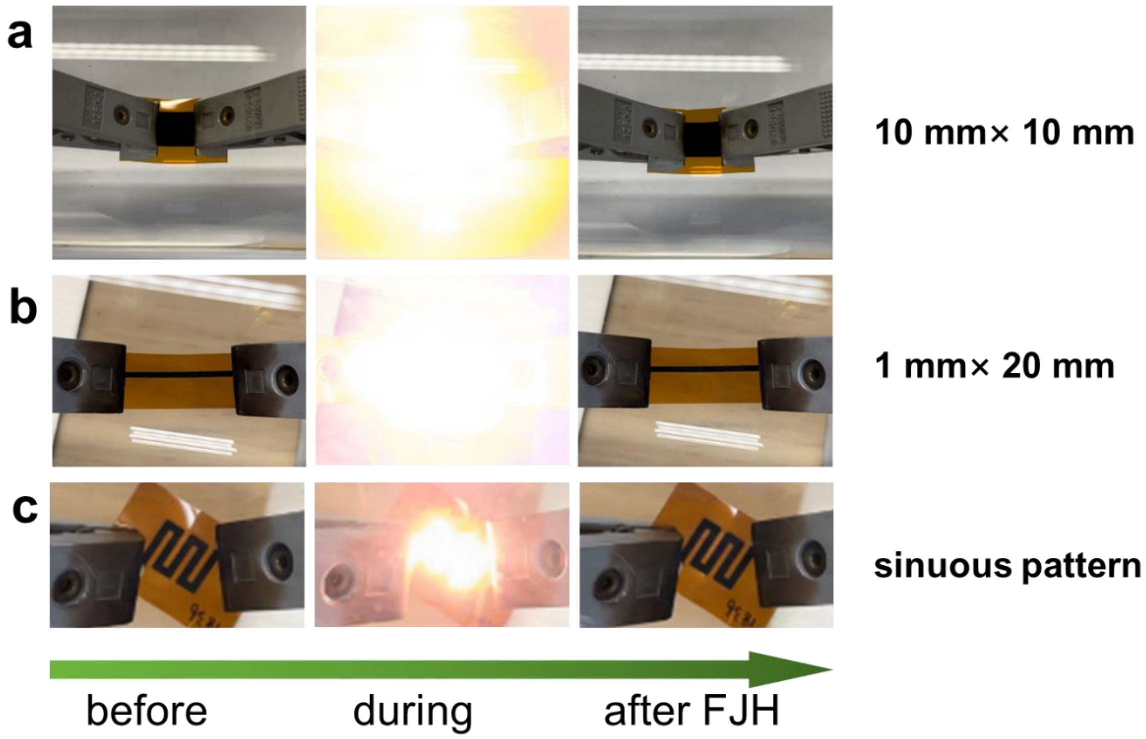

Supplementary Figure 10. FJH of LIG patterns with different shapes and dimensions. Digital photographs illustrating the FJH process of LIG patterns with dimensions of (a) 10 mm × 10 mm, (b) 1 mm × 20 mm. (c) Digital photographs of sinuous LIG patterns with 1 mm width.

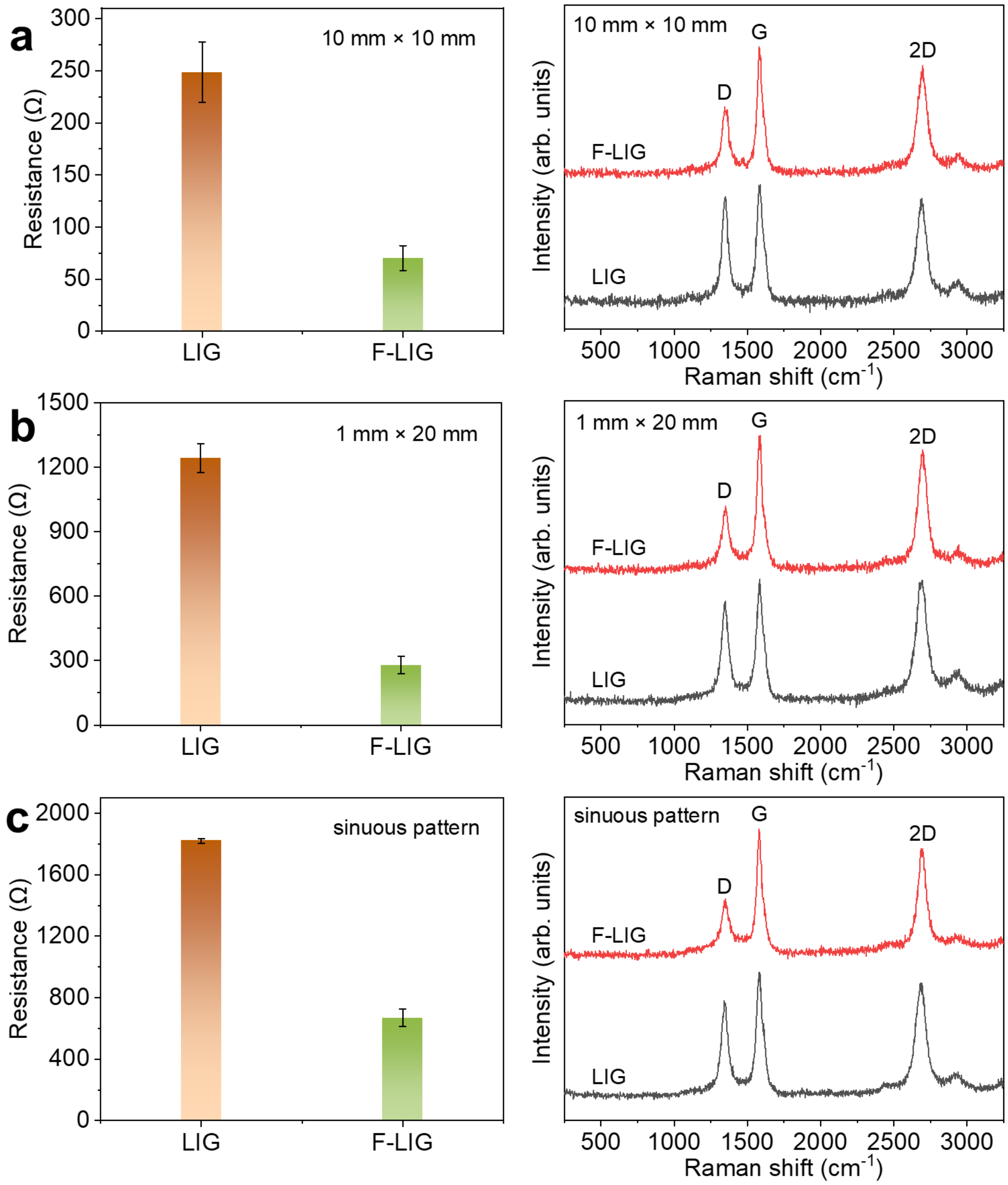

Supplementary Figure 11. Effect of FJH treatment on LIG patterns with different shapes and dimensions. Resistance and Raman spectra of LIG and F-LIG patterns with dimensions of (a) 10 mm × 10 mm, (b) 1 mm × 20 mm. (c) Resistance and Raman spectra of sinuous LIG patterns with 1 mm width. Error bars represent the standard deviation of three independent measurements.

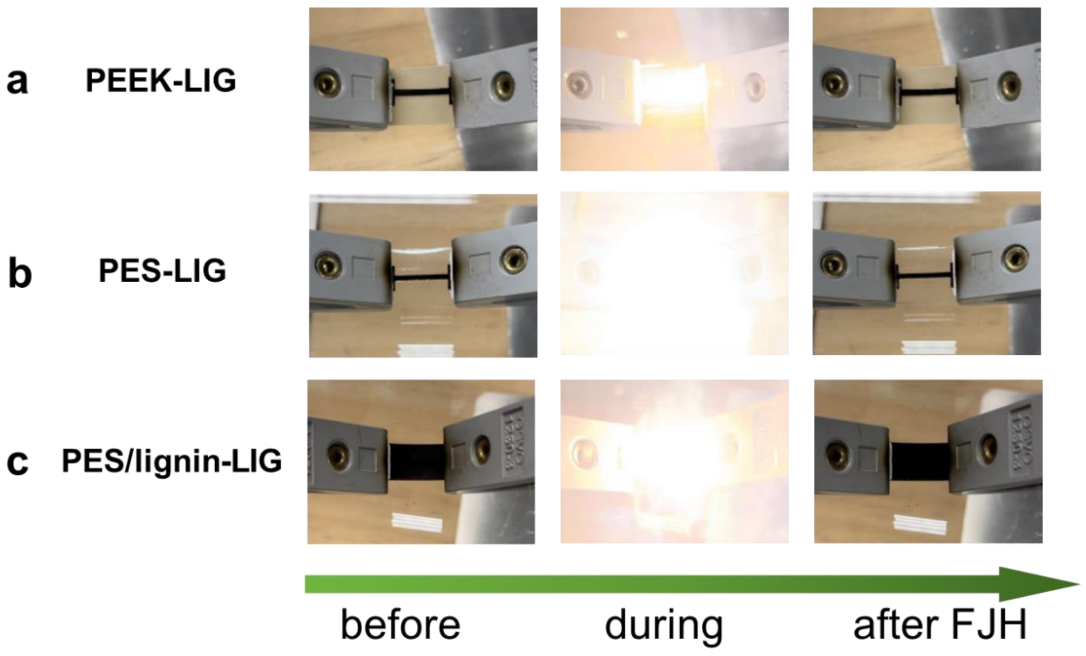

Supplementary Figure 12. FJH of LIG patterns with different substrates. Digital photographs illustrating the FJH process of (a) PEEK-, (b) PES-, and (c) PES/lignin-derived LIG patterns.

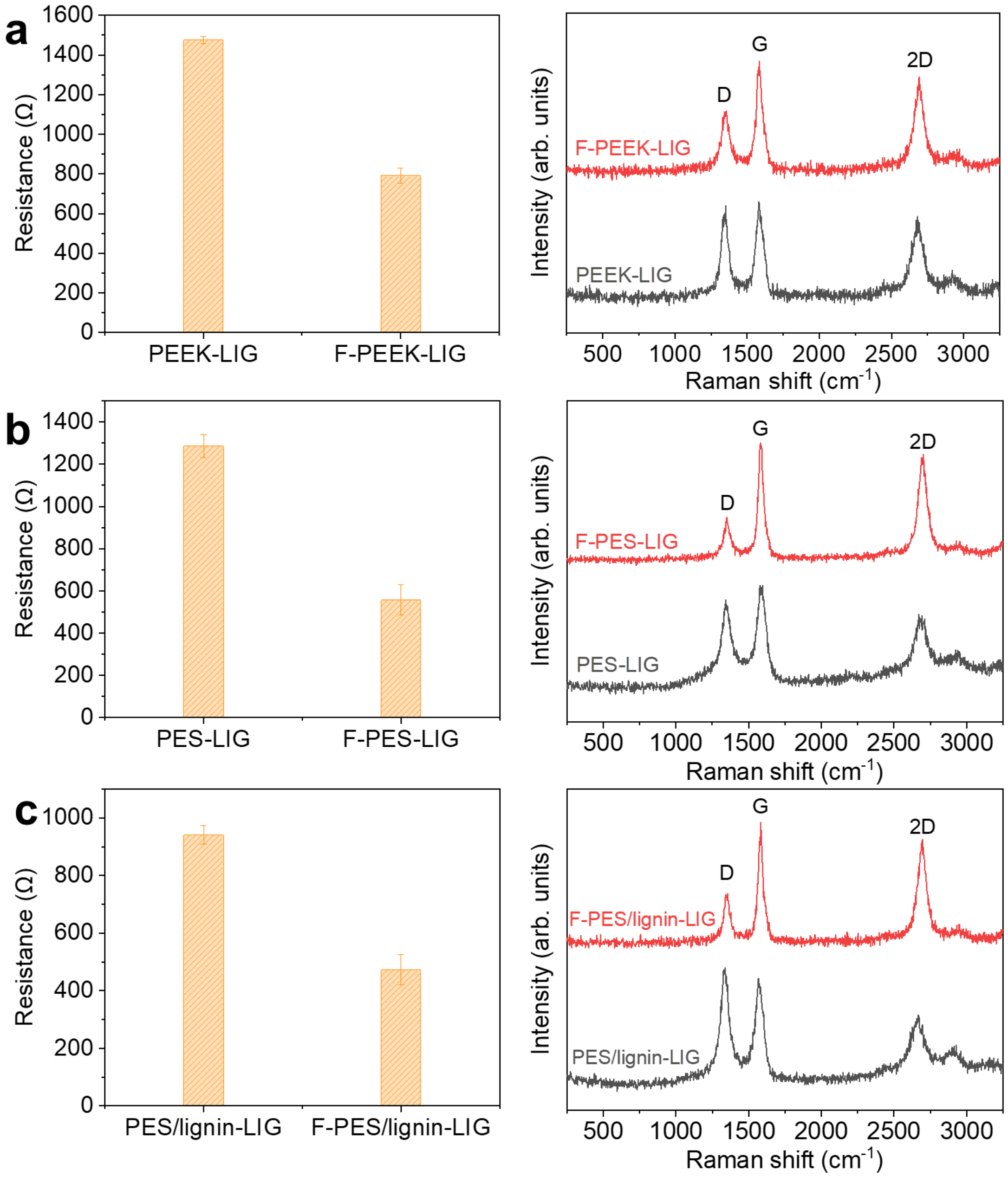

Supplementary Figure 13. Effect of FJH treatment on LIG patterns with different substrates. Resistance and Raman spectra of (a) PEEK-, (b) PES-, and (c) PES/lignin-derived LIG patterns before and after FJH process. Error bars represent the standard deviation of three independent measurements.

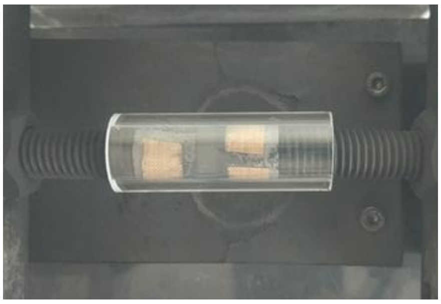

Supplementary Figure 14. Digital photographs of the quartz tube setup used for the FJH treatment of powdered samples.

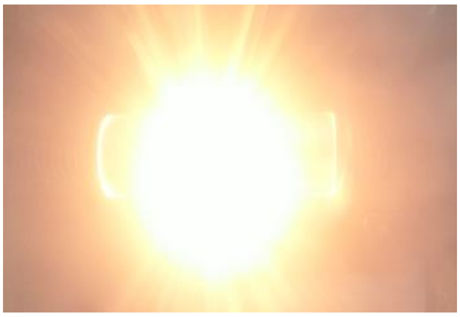

Supplementary Figure 15. Digital photographs of the FJH process of powdered LIG.

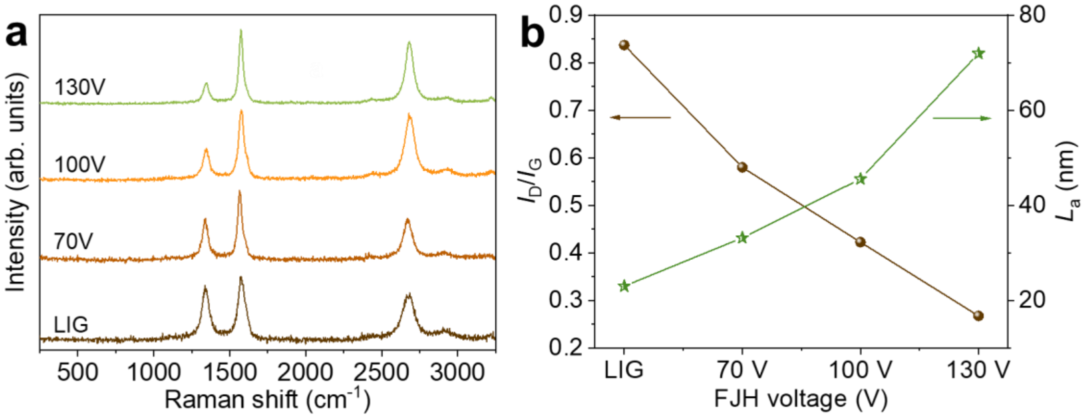

Supplementary Figure 16. Defect structure investigation of powdered LIG. (a) Raman spectra, and (b) I_D/I_G and L_a of the LIG powder samples treated under different FJH voltages.

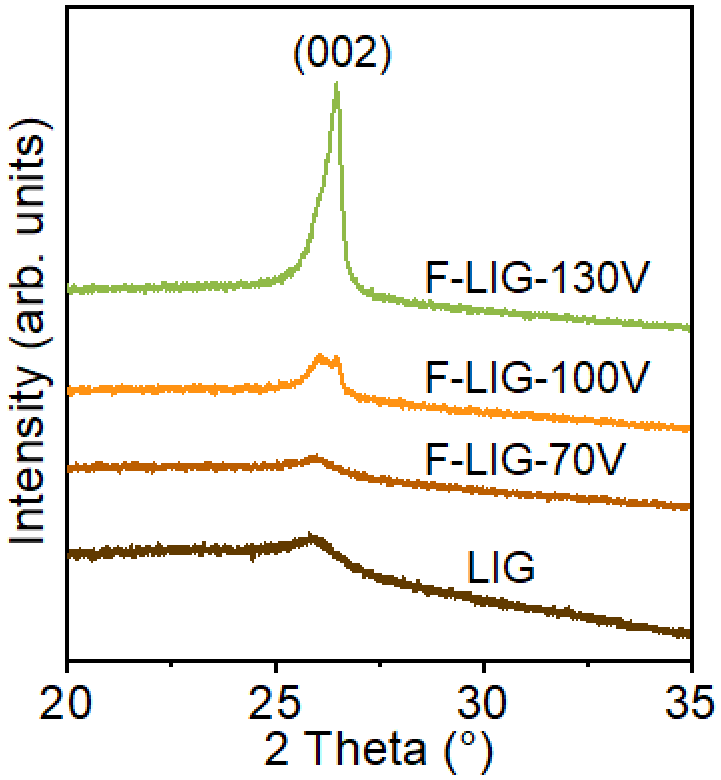

Supplementary Figure 17. XRD patterns of LIG powder samples treated under different FJH voltages.

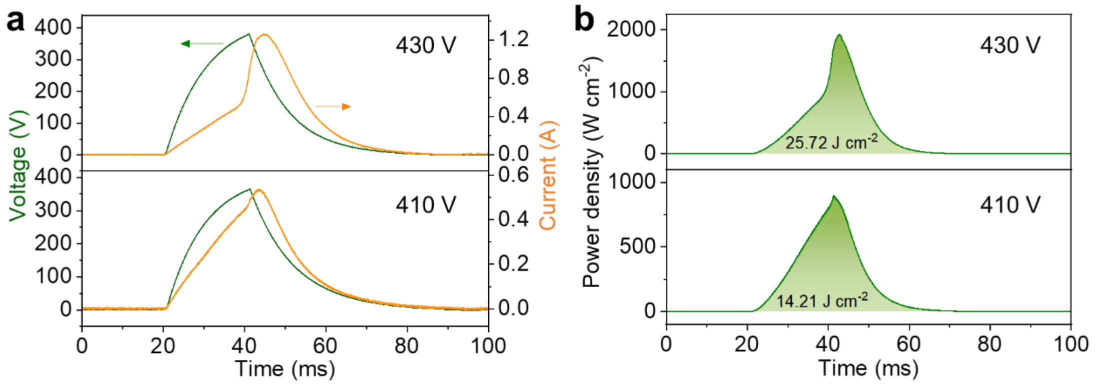

Supplementary Figure 18. FJH progress investigation of LIG patterns with dimensions of 1 mm × 20 mm. Curves of (a) voltage (green line) and current (orange line), and (b) areal power density and integrated energy density.

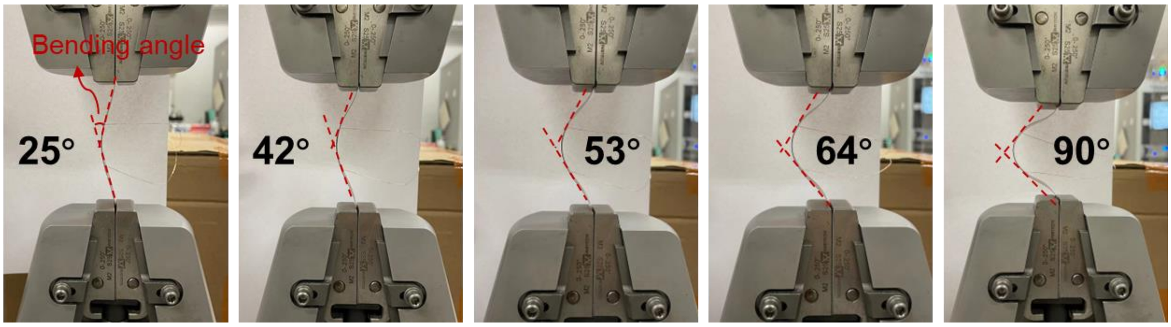

Supplementary Figure 19. Digital photographs showing different bending conditions achieved by varying the distance between the clamps.

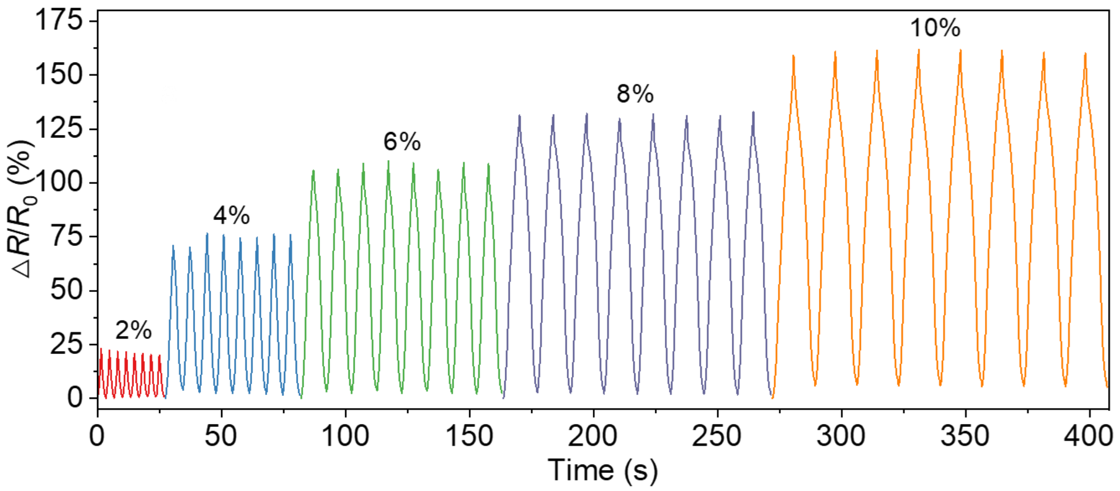

Supplementary Figure 20. Resistance variation of LIG/PDMS stretchable sensors at different strain loadings.

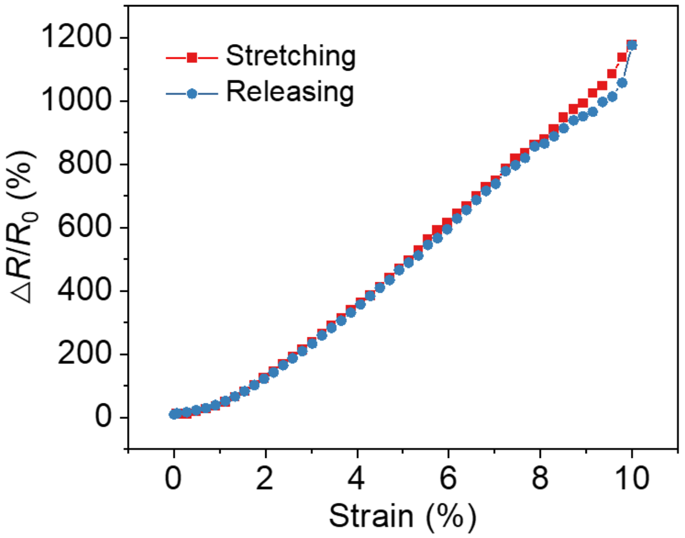

Supplementary Figure 21. Resistance variation with strain of F-LIG-H/PDMS during the stretching (red line) and releasing (blue line) up to 10% strain.

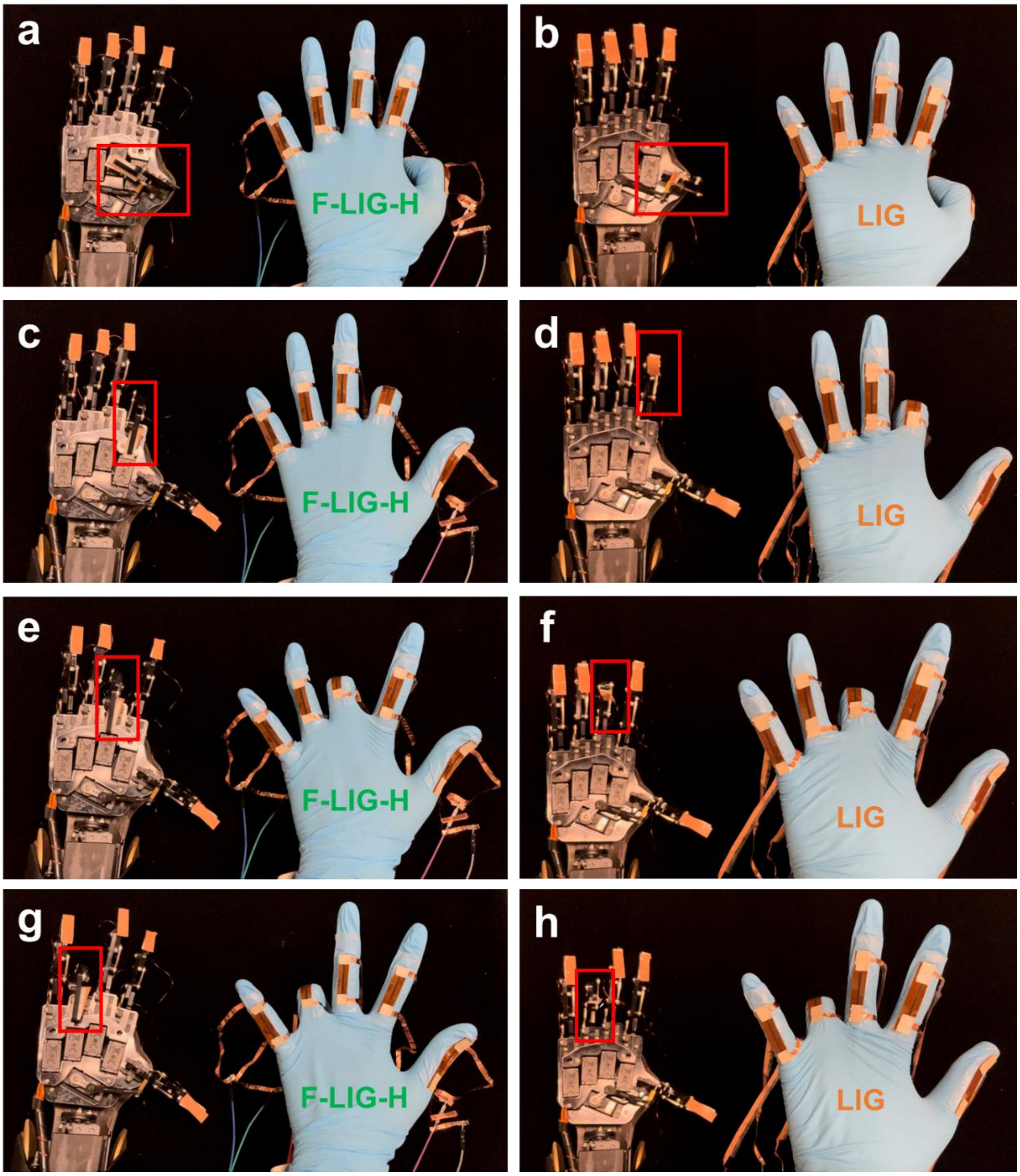

Supplementary Figure 22. Real-time control of a robotic hand. Digital photographs showing the real-time control of a robotic hand by smart gloves integrated with (a, c) F-LIG-H and (b, d) LIG sensors.

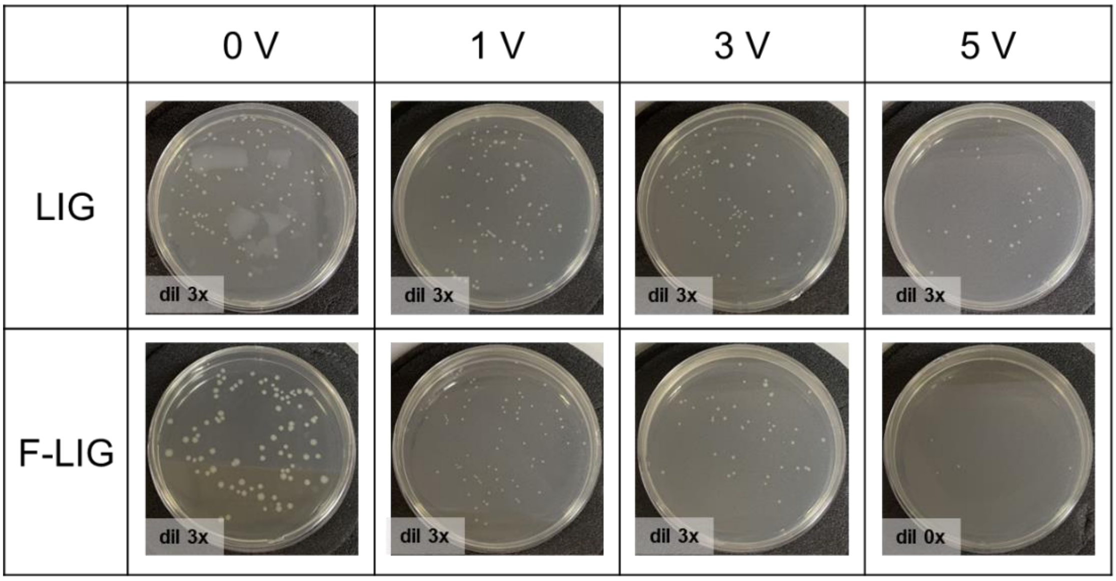

Supplementary Figure 23. Optical images of *E. coli* growth on agar plates for LIG and F-LIG films under different sterilization voltages.

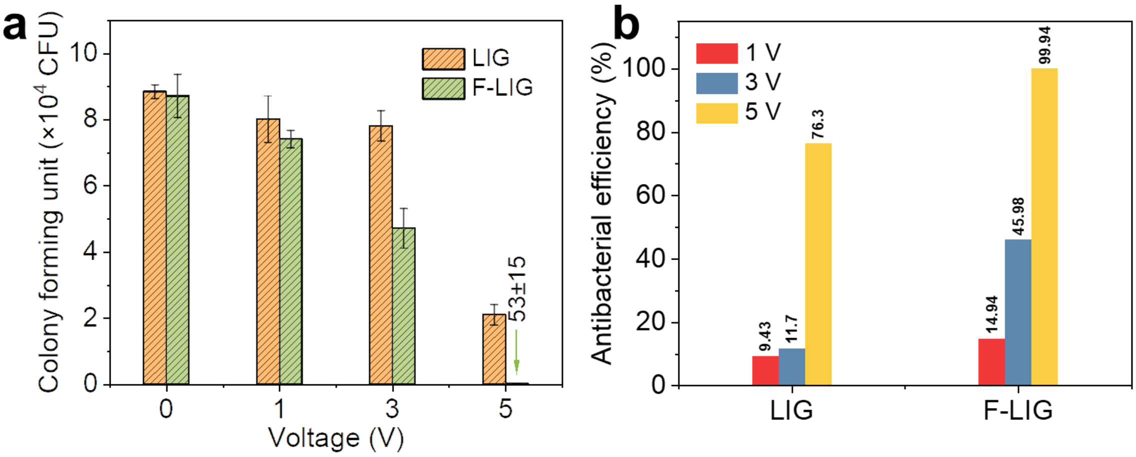

Supplementary Figure 24. Comparison of sterilization performance. (a) CFU statistics and (b) antibacterial efficiency for LIG and F-LIG films under different sterilization voltages. Error bars represent the standard deviation of three independent measurements.

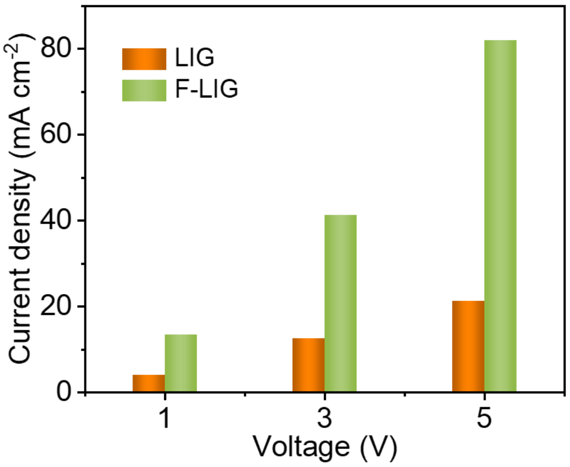

Supplementary Figure 25. Current densities passing through LIG and F-LIG films under different sterilization voltages.

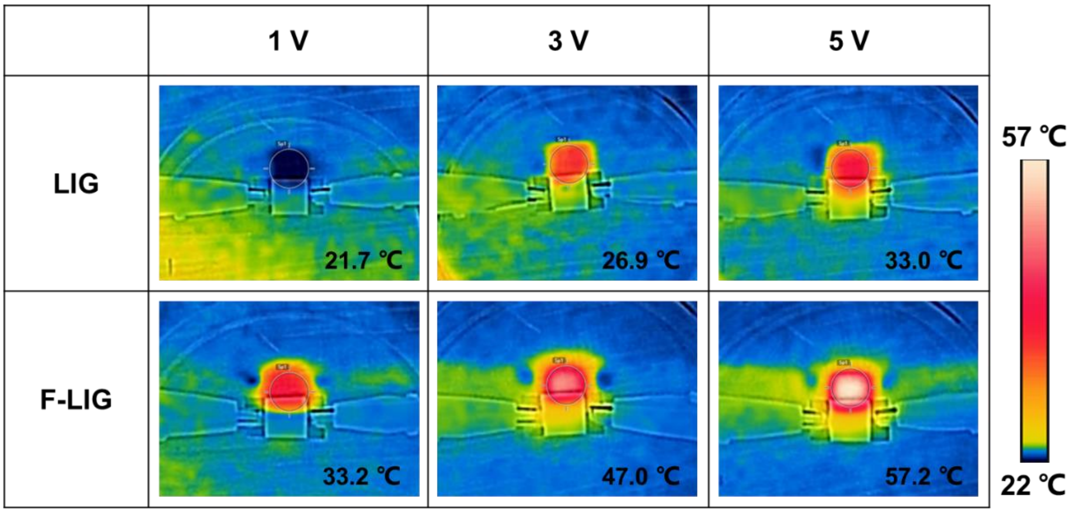

Supplementary Figure 26. Infrared thermal images of LIG and F-LIG films during the electrical sterilization process.

Supplementary Table 1. Sensitivity comparison of state-of-the-art strain sensors under low strain ranges

Sensing material	GF/strain ranges	Reference
LIG/PDMS	38 (0-10%)	[1]
LIG/PU	40 (0-10%)	[2]
Lignin-LIG/PDMS	20 (0-60%)	[3]
Wavy-LIG/PDMS	37.8 (0-32%)	[4]
$HfSe_2$/LIG-Ecoflex	46 (0-30%)	[5]
BP/LEG on SEBS	81 (0-7.5%)	[6]
MWCNTs/graphene/silicone rubber/Fe_3O_4 nanocomposites	8.43 (0-120%)	[7]
$Ti_3C_2T_x$ MXene/CNT	64.6 (0-30%)	[8]
$Ti_3C_2T_x$ MXene/AgNWs/LM-PDMS	3.22 (0-100%)	[9]
CNT-PDMS	14.5 (0-20%)	[10]
F-LIG-H/PDMS	120 (2-10%)	This work

*PU-polyurethane; BP-black phosphorus; LEG-laser-engraved graphene; MWCNTs-multi-walled carbon nanotubes; CNT-carbon nanofiber; AgNWs-Ag nanowires; LM-liquid metal

Supplementary References

1 Wang, H., Zhao, Z., Liu, P., Pan, Y. & Guo, X. Stretchable sensors and electro-thermal actuators with self-sensing capability using the laser-induced graphene technology. *ACS Appl. Mater. Interfaces* **14**, 41283-41295 (2022).

2 Dallinger, A., Keller, K., Fitzek, H. & Greco, F. Stretchable and skin-conformable conductors based on polyurethane/laser-induced graphene. *ACS Appl. Mater. Interfaces* **12**, 19855-19865 (2020).

3 Lee, C.-W. *et al.* Fabrication of laser-induced graphene-based multifunctional sensing platform for sweat ion and human motion monitoring. *Sens. Actuators, A* **334**, 113320 (2022).

4 Huang, L. *et al.* Wearable flexible strain sensor based on three-dimensional wavy laser-induced graphene and silicone rubber. *Sensors* **20** (2020).

5 Yang, H. *et al.* Stretchable strain sensor based on $HfSe_2$/LIG composite with high sensitivity and good linearity within a wide range. *Appl. Surf. Sci.* **636**, 157772 (2023).

6 Chhetry, A. *et al.* Black phosphorus@laser-engraved graphene heterostructure-based temperature–strain hybridized sensor for electronic-skin applications. *Adv. Funct. Mater.* **31**, 2007661 (2021).

7 Guo, X. *et al.* Bioinspired dual-mode stretchable strain sensor based on magnetic nanocomposites for strain/magnetic discrimination. *Small* **19**, 2205316 (2023).

8 Cai, Y. *et al.* Stretchable $Ti_3C_2T_x$ MXene/carbon nanotube composite based strain sensor with ultrahigh sensitivity and tunable sensing range. *ACS Nano* **12**, 56-62 (2018).

9 Wang, Y. *et al.* High linearity, low hysteresis $Ti_3C_2T_x$ MXene/AgNW/liquid metal self-healing strain sensor modulated by dynamic disulfide and hydrogen bonds. *Adv. Funct. Mater.* **33**, 2301587 (2023).

10 Qaiser, N. *et al.* A robust wearable point-of-care CNT-based strain sensor for wirelessly monitoring throat-related illnesses. *Adv. Funct. Mater.* **31**, 2103375 (2021).
