# Supplementary material for: Flash healing of laser-induced graphene
Source: Nat Commun. 2024 Apr 4;15:2925. doi: 10.1038/s41467-024-47341-1 (PMC10995154; doi:10.1038/s41467-024-47341-1)
Supplement: Supplementary file 3 — Description of Additional Supplementary Files [file 41467_2024_47341_MOESM3_ESM.pdf]

**Description of Additional Supplementary Files**

**File Name: Supplementary Movie 1**
**Description:** A comparison illustrating the real-time control of a robotic hand using smart gloves integrated with F-LIG and LIG sensors, respectively.

**File Name: Supplementary Movie 2**
**Description:** Manipulating a robotic hand to perform diverse gestures using the F-LIG sensors-integrated smart glove.
